# Combining EZH2 and HDAC inhibitors to target castration-resistant prostate cancers

**DOI:** 10.1371/journal.pbio.3002081

**Published:** 2023-04-27

**Authors:** Jonathan B. Coulter, Hariharan Easwaran

**Affiliations:** 1 The Brady Urological Institute, Johns Hopkins University School of Medicine, Baltimore, Maryland, United States of America; 2 Cancer Genetics and Epigenetics, Oncology, Sidney Kimmel Comprehensive Cancer Center, Johns Hopkins University School of Medicine, Baltimore, Maryland, United States of America

## Abstract

Development of resistance in castration-resistant prostate cancer (CRPC) involves epigenetic pathways involving EZH2 and HDACs. This Primer explores a PLOS Biology study showing that combined epigenetic therapy targeting these enzymes can activate cellular stress pathways, which may sensitize CRPC to both epigenetic and standard therapies.

## Teaser

Development of resistance in castration-resistant prostate cancer (CRPC) involves epigenetic pathways. A new study in *PLOS Biology* demonstrates that combined therapy targeting enhancer of zeste homolog 2 (EZH2) and histone deacetylases (HDACs) may sensitize CRPC to both epigenetic and standard therapies.

Despite advances in diagnosis and treatment, prostate cancer remains the second-leading cause of cancer-related death in men and a significant health concern. This is attributable to its high incidence (an estimated 1 in 8 men are diagnosed with prostate cancer), late stage of diagnosis and development of treatment resistance. Prostate cancer develops in the prostate gland mainly due to dysregulated androgen signaling through the androgen receptor. Androgen receptor has a critical role in regulating the growth and differentiation of prostate cells and is frequently overexpressed or mutated in prostate cancer cells. Suppression of androgen receptor signaling by androgen deprivation therapy (ADT), which involves blocking the production or activity of androgens, has been a major treatment approach for prostate cancer. However, the effectiveness of ADT is limited, and prostate cancer cells can eventually develop resistance to this therapy by finding alternative ways to activate the androgen receptor signaling pathway, leading to development of castration-resistant prostate cancer (CRPC). CRPC consequently is an advanced stage of prostate cancer with limited treatment options. Understanding the biological mechanisms involved in maintenance of CRPC and ways to directly target the resistance mechanisms are therefore critical.

**Fig 1:**
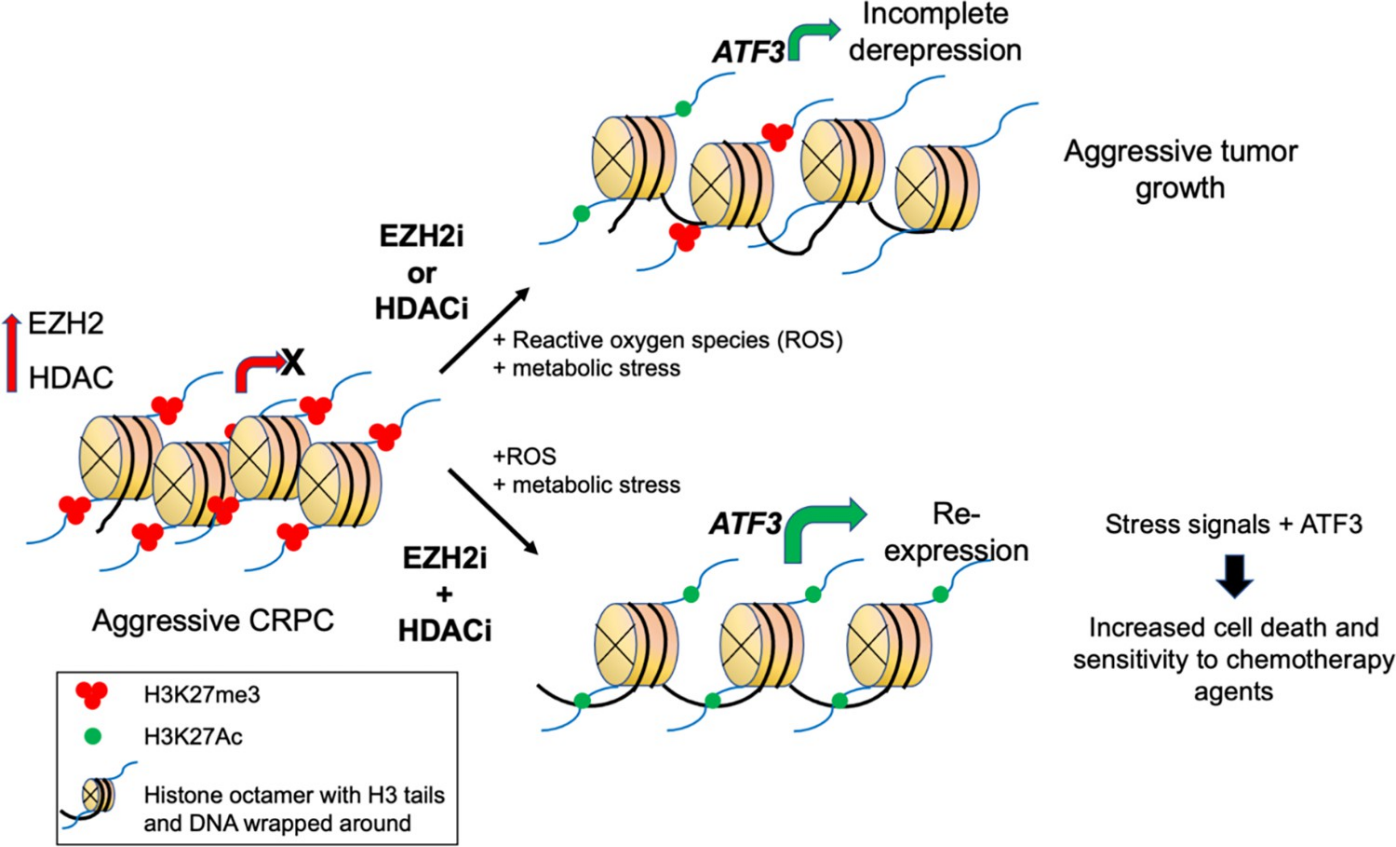
Targeting EZH2 and HDACs results in cytotoxic effects in castration-resistant prostate cancers by derepressing stress response pathways. CRPC is associated with tight suppression of large numbers of pro-tumorigenic genes due to increased expression of EZH2 and HDACs. Individual targeting of EZH2 or HDACs using small molecule inhibitors (EZH2i or HDACi) leads to incomplete derepression of tightly suppressed genes. EZH2i alone leads to only partial removal of the inactivating H3K27me3 mark by suppressing EZH2 activity; similarly, HDACi results in only partial accumulation of the activating H3K27ac mark at target genes. Combined treatment with EZH2i or HDACi resulted in the active chromatin (H3K27ac) configuration at promoter regions and deep derepression of several genes silenced in CRPCs. One such target gene relevant to resistance of CRPC is *ATF3* that is a stress response gene. ATF3 is significantly up-regulated due to *ATF3* promoter activation by combination drug treatment as well as induction of ROS and metabolic stress by these inhibitors. While EZH2i and HDACi individually also cause ROS induction and metabolic stress, it is not sufficient to activate ATF3 due to retention of inactive chromatin at the *ATF3* promoter. Up-regulation of ATF3 in the presence of ROS and metabolic signals results in killing of CRPCs. ATF3, activating transcription factor 3; CRPC, castration-resistant prostate cancer; EZH2, enhancer of zeste homolog 2; HDAC, histone deacetylase; ROS, reactive oxygen species.

Development of the CRPC state involves epigenetic reprogramming in response to persistent androgen receptor targeting, wherein the epigenetic enzyme enhancer of zeste homolog 2 (EZH2) has a critical role. EZH2 is a histone methyltransferase that complexes with polycomb repressive complex 2 (PRC2) components to catalyze the trimethylation of histone H3 lysine 27 (H3K27me3), which is associated with gene repression (Fig 1). EZH2 overexpression promotes castration resistance in prostate cancers via various mechanisms, including inducing epithelial-to-mesenchymal transition (EMT), increased stem cell potential, and androgen receptor pathway activation [1]. Besides its gene-repressive functions, EZH2 also has PRC2-independent and histone methyltransferase-independent roles in increasing androgen receptor expression and transcription factor activity. This, in turn, leads to the activation of genes associated with castration resistance [2].

In addition to EZH2, another key epigenetic enzyme important in CRPC is the class of histone deacetylases (HDACs). HDACs are enzymes that remove acetyl groups from histone proteins in chromatin, which can lead to a tighter packing of DNA and a reduction in gene expression (Fig 1). HDACs are also overexpressed in prostate cancers and required for functional androgen receptor signaling. Accordingly, HDAC inhibitors function by reducing androgen receptor activity [3]. Furthermore, interestingly both EZH2 and HDACs have non-chromatin-based mechanisms of androgen receptor activation and CRPC sustenance. Due to these roles in CRPC, small molecule inhibitors/degraders of both EZH2 and HDAC have emerged as potential therapeutic strategies in CRPC [4,5]. Individual targeting of these enzymes decreases prostate cancer cell proliferation [2,6]. However, these approaches have resulted in limited clinical success [4,5,7,8].

In a new study in *PLOS Biology*, Schade and colleagues found that targeting the epigenetic functions of both EZH2 and HDACs together had a synergistic effect in killing CRPC cells, independent of androgen receptor activity [9]. In particular, combined inhibition of EZH2 and HDACs induced expression of the key stress response transcription factor gene *ATF3*, which resulted in an acute cytotoxic effect both in vivo and in in vitro models of aggressive CRPCs. Schade and colleagues hypothesized that genes suppressed by EZH2 are incompletely derepressed upon inhibiting EZH2 alone because of sustained presence of deacetylated histones at the target gene regulatory elements (Fig 1). Thus, they tested the impact of a combination of inhibitors of EZH2 (EZH2i; GSK126) and HDACs (HDACi; Vorinostat), both of which are being used in clinical trials for CRPC as well as for other cancers. While individually these drugs were not potent, combined targeting resulted in strong inhibition of cell growth and tumor regression in in vivo CRPC models. Significantly, the drug combination had a greater up-regulation effect on previously known PRC2 signatures compared to either of the individual agents, which supports the notion that the agents were jointly derepressing a specific subset of PRC2 targets.

Schade and colleagues identified activating transcription factor 3 (ATF3) by performing expression analysis to identify up-regulated genes and pathways, ChIP-seq, and siRNA knock-down screens. *ATF3* was a direct PRC2 target that was critical for the cytotoxic effects of the combination treatment (Fig 1). ATF3 has a key role in the cellular stress response, helping cells to adapt and survive in adverse conditions. In concordance with its role as a cellular stress response gene, *ATF3* expression alone does not cause cell death but requires cell stress signals in the form of chemotherapy or stress in the context of EZH2i and HDACi. Combination epigenetic inhibition was required for strong expression of *ATF3* in the context of other stressors, such as the chemotherapy agent docetaxel. Further, the EZH2i and HDACi combination induced oxidative and metabolic stress signals. These results [9] show that combining the epigenetic inhibitors has 2 effects: inducing stress signals and causing epigenetic reprogramming, which leads to strong re-expression of *ATF3* and cell death of CRPCs. In accordance with the ex vivo and in vivo models, the authors observed that patients with prostate cancer who had higher *ATF3* levels were associated with improved overall survival. They also found that there is an inverse correlation between EZH2 and ATF3-related gene expression signatures in patients with prostate cancer. The link between stress-related pathways and cancer is intricate due to their dual roles as both oncogenic and tumor-suppressive agents. Translating the findings to the clinic will require careful analysis of stress pathways involved in the context of epigenetic therapies and the range of antitumor and oncogenic activities it may elicit in the tumor tissue environment.

Finally, combination epigenetic therapies have been a focus of research for various cancer types [10]. While EZH2i and HDACi therapies have shown effectiveness in cell line and preclinical rodent studies, they have had limited success clinically, including in prostate cancer [8,10]. Therefore, additional analysis is required to determine their efficacy in different model systems like organoids and patient-derived xenografts before clinical translation can be considered. Additionally, as mentioned earlier, EZH2 and HDACs have non-chromatin-based mechanisms of androgen receptor activation. Therefore, exploring other mechanistic modalities by which combined targeting of EZH2 and HDACs exert cytotoxic effects should be further investigated. Finally, this study indicates that combination epigenetic treatment and consequent activation of ATF3 can sensitize CRPCs to chemotherapy, which is an avenue that should be further explored to improve treatment approaches. In summary, with further follow-up studies, the hope is that such combination epigenetic therapies may lead to better outcomes for those with CRPC.

## Abbreviations

DT: androgen deprivation therapy
ATF3: activating transcription factor 3
CRPC: castration-resistant prostate cancer
EMT: epithelial-to-mesenchymal transition
EZH2: enhancer of zeste homolog 2
HDAC: histone deacetylase
PRC2: polycomb repressive complex 2
